# Digestibility, Passage Rate, Growth, and Digesta Properties in Windsnyer Pigs Fed Increasing Potato Hash Silage

**DOI:** 10.3390/ani15243596

**Published:** 2025-12-15

**Authors:** Cyprial Ndumiso Ncobela, Arnold Tapera Kanengoni, Michael Chimonyo

**Affiliations:** 1 Department of Agriculture, Faculty of Applied and Health Sciences, Mangosuthu University of Technology, P.O. Box 12363, Jacobs 4026, South Africa; 2 Production Animal Studies Department, Faculty of Veterinary Sciences, University of Pretoria, Private Bag X04, Onderstepoort 0110, South Africa; atkanengoni@gmail.com; 3 Faculty of Science, Engineering and Agriculture, University of Venda, Private Bag X5050, Thohoyandou 0950, South Africa; michael.chimonyo@univen.ac.za

**Keywords:** feed intake, fermentation, fibre, gut, maize cob, pig, potato hash silage

## Abstract

The study was conducted to determine the apparent total tract digestibility, the rate of digesta passage, growth performance, and physicochemical properties of the digesta of Windsnyer pigs fed on increasing levels of potato hash silage. Diets were formulated to contain 0, 80, 160, 240, 320, and 400 g potato hash silage/kg of diet. Pigs were randomly assigned to six diets according to a completely randomised design. Six pigs were allocated to each dietary treatment. All diets were blended with chromic oxide (Cr_2_O_3_) to calculate apparent total tract digestibility and rate of passage. Pigs were fed diets containing different levels of potato hash silage for 5 d, following 7 d of adaptation. Thereafter, pigs were subjected to a 35-day growth performance experiment. Thirty-six pigs were slaughtered and eviscerated to determine digesta characteristics and gut compartment weights. There was a positive correlation (*p* < 0.05) between mean retention time and each of digestibility of dry matter, organic matter, and crude protein. There was a quadratic increase (*p* < 0.05) in digestibility of organic matter, crude protein, fibre and feed intake as dietary levels of potato hash silage increased. Average daily gain and gain-to-feed ratio decreased linearly (*p* < 0.05) in pigs as levels of potato hash silage increased. The swelling capacity (SWC) of the digesta from ileum decreased linearly (*p* < 0.05) as dietary levels of potato hash silage increased. The SWC of the digesta from the caecum increased linearly (*p* < 0.05) as dietary levels of potato hash silage increased. The water holding capacity (WHC) of the stomach digesta decreased linearly (*p* < 0.05) as dietary levels of potato hash silage increased. The WHC of the digesta from the ileum and caecum displayed negative quadratic responses (*p* < 0.05) as dietary levels of potato hash silage increased. The WHC of digesta from the proximal colon showed a linear decline (*p* < 0.05) as dietary levels of potato hash silage increased. The WHC of digesta from the distal colon increased linearly (*p* < 0.05) as dietary levels of potato hash silage increased. The results indicated that Windsnyer pigs can effectively utilise potato hash silage in diets until the 240 g/kg inclusion level beyond which total tract digestibility and feed intake are compromised.

## Simple Summary

Indigenous pigs, such as the Windsnyer found in Southern Africa, are important for smallholder farmers because they are hardy and can survive on low-quality feed resources. However, these pigs are slowly disappearing because they are often seen as less productive than modern breeds. One way to make them more valuable is by using local feed materials that are cheap and widely available, such as potato hash, which is a waste product from potato processing. This study tested whether Windsnyer pigs could benefit from eating silage made from potato hash mixed with maize cobs. We found that pigs were able to digest and use the nutrients well when the potato hash silage was included up to a certain level in the diet. At 240 g/kg, the pigs ate more feed and digested more nutrients, but at higher levels, their growth and feed use declined. This means that potato hash silage can be safely used in pig diets, reducing waste and feed costs for farmers, while also supporting the conservation of local pig breeds. The results provide a sustainable feeding option that can help improve food security and livelihoods in rural communities.

## 1. Introduction

Sustainability of smallholder pig production in Southern Africa can be strengthened if resource-limited farmers utilise hardy pig genotypes that can perform on less nutrient-dense and more variable feed resources [1]. Indigenous breeds such as the Kolbroek, Mukota, and Windsnyer pigs are well suited to these systems because of their adaptability, resilience, and lower nutrient requirements compared with imported commercial breeds [2]. Windsnyer pigs, which possess dark skin, compact bodies, and long snouts, are particularly hardy; however, their numbers have declined substantially, raising concerns about their long-term conservation [2]. Their declining popularity stems partly from historical biases against extensive production systems traditionally associated with indigenous breeds and the continued promotion of imported fast-growing genotypes by commercial organisations [3]. Preserving and improving the productivity of Windsnyer pigs is therefore important for maintaining locally adapted genetic resources that support resilient smallholder production. In response to this need, the Agricultural Research Council (ARC) of South Africa operates a Germplasm Conservation and Reproductive Biotechnologies programme aimed at conserving purebred Windsnyer pigs for research, breeding, and production.

Beyond their genetic value, Windsnyer pigs are known for their adaptability to harsh environments, tolerance to endemic diseases, and strong ability to utilise fibrous agricultural by-products [4]. This makes them particularly relevant for smallholder systems where conventional feed ingredients are expensive. One such underutilised resource is potato hash, a by-product generated during the processing of potatoes (*Solanum tuberosum*). Potato hash is produced in large quantities in some regions and thus represents a potentially affordable and sustainable feed ingredient for smallholder pig farmers. Its nutrient profile—rich in metabolizable energy (11.2–11.4 MJ/kg DM) and starch (700–704 g/kg DM), containing moderate crude fibre (360–370 g/kg DM), and low crude protein (105–110 g/kg DM)—suggests that it could serve as an effective energy source in pig diets [5]. However, its high moisture content accelerates spoilage, limiting its use in fresh form.

Ensiling is a practical and low-cost preservation strategy for stabilising potato hash, but its inherently high moisture content requires mixing with absorbents to achieve optimal dry matter (DM) levels for fermentation [6]. This creates a composite silage product whose nutritional behaviour cannot be predicted from the raw materials alone. Ensiling alters substrate availability, generates fermentation acids, and modifies physical attributes such as water-holding capacity and particle structure [5]. Consequently, the digestibility, fermentability, and passage characteristics of potato hash silage may vary depending on its composition and inclusion level in the diet.

The physicochemical properties of feed, including water-holding capacity (WHC), viscosity, and swelling capacity (SWC), play significant roles in determining nutrient digestibility, digesta movement through the gastrointestinal tract, and the efficiency of microbial fermentation [7].

Potato hash contains both starch and fibre components [5] that may influence these properties in a level-dependent manner. For example, higher inclusion levels may increase digesta bulk or viscosity, altering the rate of passage, while lower levels may enhance energy supply without markedly affecting gut transit [8]. Since Windsnyer pigs have demonstrated a strong capacity to utilise fibrous by-products [5], they may respond differently to potato hash silage compared with commercial breeds; however, their digestive responses to this specific ingredient remain unknown. Understanding how increasing dietary levels of potato hash silage influence digestibility, rate of passage, and digesta characteristics is therefore essential before making dietary recommendations for smallholder production systems. Graded inclusion testing is necessary to identify both beneficial responses and potential thresholds beyond which nutrient utilisation or gut function may be compromised. The objective of the present study was thus to determine the effects of increasing levels of potato hash silage on apparent total tract digestibility (ATTD), rate of passage, growth performance, and physicochemical properties of the digesta of growing Windsnyer pigs. It was hypothesised that increasing dietary inclusion of potato hash silage would lead to corresponding increases in ATTD of nutrients, rate of passage, and improvements in key digesta characteristics.

## 2. Materials and Methods

### 2.1. Study Site

The study was conducted at the ARC, Animal Production Institute, Irene, South Africa. The institute lies at 25°34′0″ S and 28°22′0″ E and is approximately 1526 m above sea level. The average annual temperature is 18.7 °C.

### 2.2. Animals, Experimental Design and Diets

The use and care of the experimental animals were approved by the Animal Research Committee (ARC), Animal Production Institute Ethics Committee, South Africa (Reference number: APIEC16/015). A total of 36 clinically healthy male growing Windsnyer pigs with an average initial body weight of 22 ± 4.43 kg (mean ± standard deviation), aged between three and four months were selected from the indigenous pig herd of the ARC and used in the current study. There was no significant difference in the initial body weight. The Windsnyer pigs were moved from the indigenous pig herd where they were housed in groups, to a trial facility where they were individually penned in 1.0 × 0.9 m pens with slatted concrete floors. Each pen was equipped with a single-space feeder and a low-pressure nipple drinker. The temperature and relative humidity in the experimental house were maintained at 24.5 °C ± 1.9 °C and 62.7% ± 15.07%, respectively (mean ± standard deviation). The temperature and relative humidity were recorded every 30 min with log tags. The 36 pigs were randomly assigned to experimental diets containing 0, 80, 160, 240, 320, and 400 g potato hash silage/kg of diet according to a completely randomised design. The total mixed diets with increasing levels of potato hash silage were formulated to meet nutritional requirements of slow-growing pigs [9], using the Winfeed software programme [10]. The experimental diet compositions are shown in Table 1. The six dietary treatments were each assigned to six pigs, with each pig serving as an experimental unit. The pigs were adapted to the feed with chromic oxide (Cr_2_O_3_) for 7 d before data collection for passage rate and ATTD determinations. After the digestibility study, a 35-day growth performance study was undertaken.

### 2.3. Collection, Ensiling and Mixability of Potato Hash

Fresh potato hash was collected from a local food producing company (Simba, Johannesburg, South Africa) and brought to the ARC institute for ensiling. The potato hash, which had a dry matter (DM) content of 152 g/kg upon receipt, was blended with ground maize cobs at a ratio of 7:3 (potato hash/maize cobs) to increase the DM concentration to between 250 and 400 g/kg prior to ensiling. The maize cobs were initially ground through a 5 mm hammer mill screen, after which the combined potato hash–maize cob mixture was thoroughly blended and compacted into 210 L drums lined with polyethylene plastic bags. The bags were tightly knotted to prevent air penetration, and the drums were sealed with rubber lids to protect the silage from rodents. Drums were stored at ambient temperatures ranging from 22 °C to 29 °C. Silage drums were opened weekly only during diet mixing to limit oxygen exposure, and the silage was fed to pigs within 6 months of production.

Because chromium oxide (Cr_2_O_3_) was used as an indigestible marker, special attention was given to the mixability and particle size compatibility of the potato hash–maize cob silage with the basal diet ingredients. After ensiling, the silage was re-processed through a 5 mm hammer mill screen, producing a medium-coarse particle size appropriate for homogeneous mixing within a complete diet. For the basal ingredients, corn grain was milled through a 3 mm screen, while soybean meal was supplied already finely ground (approximately 3 mm). The resulting similarity in particle size among the silage, corn, and soybean meal facilitated even distribution of the marker. Two grams per kilogram of Cr_2_O_3_ were thoroughly blended with the experimental diets, and all ingredients were accurately weighed and mixed in a horizontal ribbon mixer for 10 min to ensure consistent marker incorporation and uniform dietary composition across treatments.

### 2.4. Measurements of Passage Rate and Apparent Total Tract Nutrient Digestibility

Before offering the pigs feed containing Cr_2_O_3_, faecal samples from each pig were collected at 07:30 and were denoted as blank samples. It was ensured that pigs were not hungry before they were offered the marked feed. Pigs were offered 1 to 1.2 kg of unmarked feed at 08:00 in the morning. They were, afterwards, supplemented with at most 1 kg of marked feed at 17:00 depending on each one’s ability to finish feed offered in the morning. The feed was offered to ensure it was 80% to 90% of ad libitum. The moment at which the pig first inserted its head into the feeder was recorded as the initial time of feed consumption. The pigs were visually monitored for the first appearance of marker every hour starting from 12 h of consumption of marked meal. Thereafter, the time to which first appearance of the marked faeces was recorded. Subsequently, the urine-free fresh faecal samples were collected from the floor pen in 4 h intervals from pens for 48 h to determine the chromium concentration. Only freshly defeated faecal samples were collected.

After that, all faeces, in each pen, defecated from 07:30 a.m. to 19:30 p.m. were collected once a day for 5 d for the estimation of total faecal weights and ATTD. The collected faecal samples were immediately stored at −20 °C after collection until analysis. At the end of the collection period, samples were thawed overnight and dried at 60 °C for 24 h before analyses. For the rate of passage determination, faecal samples for each pig at a given collection time were dried separately and were analysed for chromium concentration. For ATTD determination, the 5-day faeces for each pig were combined and mixed after the drying period and then representative samples were analysed.

### 2.5. Calculation of Rate of Passage, Faecal Scoring and Digestibility

The transit time (TT) was measured as the time between consumption of marked meal and first appearance of the marker in the faeces. The mean retention time (MRT) (in hours) in the entire gastrointestinal tract was calculated by a non-compartmental method. The term non-compartmental is used when there is no attempt made to link the MRT to the anatomical or physiological compartments. It is a suitable method when predicting total tract MRT [11]. This method does not require specific objectives and hypotheses regarding response patterns of marker excretion over time. The MRT of the digestive tract (between ingestion of the marked feed and excretion in faeces) was calculated according to equation described by Faichney [12] for total cumulative feed marker collection:

$$MRT(in\;hours)=\sum_{i=1}^{n}m_{i}t_{i}$$

where *t_i_* is the time (in hours) between the sampling interval and *m_i_* is the concentration of marker excreted in the faeces.

The faecal scoring in each pig for 5 d was visually scored as: 1 = watery, 2 = semi-watery, 3 = normal, 4 = semi-dry, 5 = dry.

The ATTD of DM (DMD), organic matter (OMD), CP (CPD), neutral detergent fibre (NDFD), and acid detergent fibre (ADFD) was calculated based on chromium concentrations in feed and faeces. The formula used for the calculation of apparent total tract nutrient digestibility was:

$$ATTD=100-\left(\frac{\%Nutrient\;faeces}{\%Nutrient\;feed}\times \frac{\%Indicator\;feed}{\%Indicator\;faeces}\right)$$

where ATTD is the apparent total tract digestibility as a coefficient; nutrient faeces is the percentage of nutrient in the faeces; nutrient feed is the percentage of nutrient in the feed; indicator feed is the percentage of chromium in the feed; indicator faeces is the percentage of chromium in the faeces.

### 2.6. Measurements of Feed Intake and Growth Performance

The feed intake and growth performance experiment were carried out over 35 days and water and feed were offered ad libitum. Average daily feed intake (ADFI) was calculated as feed consumed minus orts and spillage in each week divided by seven. Plastic trays were placed underneath each feeder in the pen to collect spillages. Feed spillages and orts were subtracted from the total weight of feed given to pigs each week. Feed given, feed spillages and orts were weighed using a digital platform scale (Min 0.1 kg to max. 300 kg) from SCALERITE SCALES, Pretoria, South Africa. Pigs were weighed weekly in the morning before feeding, and the average daily gain (ADG) was calculated for each week. ADG was calculated as final weight minus the initial weight divided by seven. The pigs were weighed using the Livestock Scales—Micro T7E Pig Scale (Premier Scale Services) from SCALERITE SCALES, Pretoria, South Africa with a maximum capacity of 600 kg and a minimum division of 0.2 kg. The gain–feed ratio was calculated as ADG divided by ADFI.

### 2.7. Slaughtering of Pigs and Measurements of Digesta pH, Digesta Compartment and Weights

All 36 pigs were transported to an abattoir located approximately 1.5 km from the trial facility at 08:30 h. The pigs were handled in accordance with standard abattoir protocols, including ante-mortem inspection by qualified personnel. Humane endpoints were not required during the trial as no adverse health conditions were observed that warranted early euthanasia. However, all pigs were monitored daily for signs of distress or illness by trained personnel throughout the study period. Slaughter was a planned and approved experimental endpoint for the purpose of collecting gastrointestinal digesta and organ measurements. Prior to slaughter, pigs were assessed for health status to ensure minimal suffering and suitability for the procedure. The slaughter process involved stunning using an electrical stunner set at 220 volts and 1.8 amps with a current duration of 6 s, followed by immediate exsanguination within 10 s to ensure rapid and humane death. No pigs died prior to slaughter. The entire study, including animal handling and slaughter, was approved by the ethics committee, which reviewed and accepted the use of slaughter as the experimental endpoint in line with scientific and ethical standards. No analgesics or anaesthetics were required prior to slaughter as the stunning procedure ensured unconsciousness.

After slaughtering, pigs were dehaired and eviscerated. The gastrointestinal tract (GIT) (from oesophagus to rectum) was separated from the carcass. The GIT was ligated at the proximal and distal ends of each compartment by double-tying and cutting to prevent digesta flow between segments. Five gut compartments namely stomach, ileum, caecum, proximal and distal colon were used. The ileum was determined as the section, which covers about 100 cm of small intestine before the ileo-caecal ostium [13]. The caecum and colon were considered as part of the gut entering the pelvic cavity and reaching the rectum part of gut that is attached to the anus. The colon compartment was separated into two equal parts namely proximal and distal colon. About 20 g of digesta sample was collected from each compartment and put into 50 mL plastic bottles. Immediately thereafter, pH was assessed using a Crison 52 02 glass pH electrode from Centurion, South Africa. The digesta sample was then frozen at −20 °C pending analyses of physicochemical properties. The weights of the compartments and digesta were measured using a digital scale (MZP Precise Electronic Balance 50 kg/0.1 g digital weighing scales) from Centurion, South Africa. The weights of stomach, small intestine, caecum and colon were determined by dividing the weights of the gut compartment by the slaughter body weight. The weights of the gut compartments and digesta were scaled to account for the differences in body weights.

### 2.8. Chemical and Physicochemical Analyses of the Diets, Faeces and Digesta

Samples of all diets, faeces and digesta were collected and analysed in triplicate (Table 2). Procedures from AOAC [14] were used to determine DM (method 2001.12), ash (method 942.05), crude protein (CP, method 990.03) and ether extract (EE, method 963.15). Neutral detergent fibre (NDF) and acid detergent fibre (ADF) were determined according to ANKOM Technology Method as explained by Van Soest et al. [15]. The NDF content was assayed using heat stable α-amylase (Sigma A3306; Sigma Chemical Co., St. Louis, MO, USA). Gross energy of potato hash silage and experimental diets were determined with bomb calorimetry (MS-1000 modular calorimeter; Energy Instrumentation, Centurion, South Africa). Calcium and phosphorus contents were determined by atomic absorption flame spectroscopy, according to method 6.5.1 of AOAC [14]. The bulk density of the diets and potato hash silage was measured using the water displacement method, as described by Kyriazakis and Emmans [16]. Briefly, 50 g of feed were weighed and placed into a 250 mL volumetric flask containing 100 mL distilled water in a water-bath at 37 °C. After mixing, an additional 50 mL of water were added, and the contents were allowed to equilibrate for 15 min. Additional 50 mL water were then added. After allowing 15 min to equilibrate, the flask was filled by adding water. The total amount of water contained in the flask was subtracted from 250 mL. The WHC of diets was measured using the centrifugation method as described by Whittemore et al. [17]. Briefly, about 0.5 g of sample were measured into a known weight of a 50 mL plastic centrifuge tube and 25 mL distilled water was added. The tubes were sealed firmly and shaken intermittently for 24 h. They were then centrifuged at 6000× *g* for 15 min at 20 °C. The supernatant was discarded, and fresh weight of the sample was determined. The samples were then dried at 103 °C for 20 h. The weights of the fluid retained were calculated from the difference between fresh sample and dried sample. The weight of the fluid retained was divided by the weight of the dried sample to determine WHC, which was expressed in g water/g of dry material. For digesta characteristics, the analysis of digesta WHC was performed on wet materials. A SWC was measured according to Canibe and Bach Knudsen [18]. Samples of the experimental diets and potato hash silage, weighing 2 g were transferred into 15 mL measuring plastic tubes. A solution of 9 g/L NaCl containing 0.2 g/L NaN_3_ was added to a final volume of 10 mL where samples were incubated at 39 °C in a water shaking bath overnight. After 16 h, the shaker was stopped, and samples were left in the water for 1 h before being taken out to measure the volume occupied by the experimental diets and potato hash silage. The SWC of the digesta was analysed on freeze-dried materials. For passage rate and digestibility, the concentration of chromium in the representative diets and faeces was determined using inductively coupled plasma atomic emission spectroscopy (Optima 5300 DV Spectrometer; Perkin Elmer, Shelton, CT, USA).

### 2.9. Statistical Analyses

Analysis of the data was performed using the Statistical Analysis System software (SAS, Version 9.1) A PROC MEANS of SAS [19] procedure was used to determine mean and standard error for potato hash silage inclusion level against nutrient utilisation, rate of passage, growth performance and physicochemical properties of the digesta. Normality of the data was assessed using the Shapiro–Wilk test. An orthogonal polynomial contrast of SAS [19] was used to determine linear, quadratic, and cubic trends between inclusion levels of ensiled potato hash against nutrient utilisation, rate of passage and physicochemical properties of the digesta. Pearson’s correlation coefficients were estimated to determine the relationship between the rate of digesta passage and nutrient digestibility. Statistical significance was established at *p* ≤ 0.05

## 3. Results

### 3.1. Apparent Total Tract Digestibility of Nutrients and Rate of Digesta Passage

The rate of marker recovery was not different across all inclusion levels and ranged from 90% to 95%. As inclusion levels of potato hash silage increased, the durations between defaecation episodes were shorter. Transit time was positively correlated with DMD, OMD, CPD, and NDFD (*p* < 0.05) as shown in Table 3. There was also a positive correlation (*p* < 0.05) between MRT and each of DMD, OMD, and CPD. Effects of dietary levels of potato hash silage on apparent total tract nutrient digestibility and rate of digesta passage in growing Windsnyer pigs are shown in Table 4. The DMD, OMD, CPD, and NDFD increased quadratically as the inclusion of level of potato hash silage increased from 0 to 240 g/kg DM and quadratically decreased after 240 g/kg DM as shown in Table 4. The maximum digestibility coefficients for DM (0.92), OM (0.93), CP (0.84), and NDF (0.75) were at the 240 g potato hash/kg diet inclusion level. The inclusion level of potato hash silage had no effect on faecal scores. There was a linear decrease (*p* < 0.05) in TT with increase in inclusion levels of potato hash silage. The MRT had a negative quadratic response (*p* < 0.05) as inclusion levels of potato hash silage increased with the highest retention time of 23.8 h at the 80 g potato hash/kg diet inclusion level. There was a linear increase (*p* < 0.05) in fresh and dry faecal weight as inclusion levels of potato hash silage increased.

### 3.2. Feed Intake and Growth Performance

The growth performance parameters of the pigs fed on different inclusion levels of potato hash silage are shown in Table 5. The ADFI had a quadratic increase (*p* < 0.05) to dietary inclusion level of potato hash silage with the maximum intake (1.92 kg) at the 240 g potato hash/kg diet inclusion level. There was a linear decrease (*p* < 0.05) in each of the ADG, gain-to-feed ratio and final body weight with increasing levels of potato hash silage.

### 3.3. Weights of Compartments of the Gastrointestinal Tract and Digesta Characteristics

The effects of dietary levels of potato hash silage on scaled digesta and gut compartment weights are shown in Table 6. There were linear increases (*p* < 0.05) in scaled compartment weights of the stomach and colon with an increase in inclusion levels of potato hash silage. There was a linear increase (*p* < 0.05) in scaled digesta weight from the colon with increasing levels of potato hash silage. The effects of dietary levels of potato hash silage on digesta pH in the GIT compartments are shown in Table 7. There was a linear increase (*p* < 0.05) in ileum digesta pH as inclusion levels of potato hash silage increased. The pH of the digesta from the distal colon decreased linearly (*p* < 0.05) as inclusion levels of potato hash silage increased. The effects of inclusion levels of potato hash silage on SWC and WHC of the digesta on Windsnyer pigs are shown in Table 8. The SWC of the digesta in the stomach increased quadratically (*p* < 0.05) as inclusion levels of potato hash silage increased with the highest SWC value (3.45 mL/g DM) at the 240 g potato hash silage inclusion level. The SWC of the digesta from ileum decreased linearly (*p* < 0.05) when pigs were fed increasing levels of potato hash silage. There was a linear increase (*p* < 0.05) in SWC of the digesta from the caecum with increase in inclusion levels of potato hash silage. The WHC of the digesta in the stomach decreased linearly (*p* < 0.05) as the dietary inclusion of potato hash silage increased

The WHC of the digesta from ileum and caecum decreased quadratically (*p* < 0.05) as inclusion levels of potato hash silage increased, with the minimum WHC values (3.10 g water/g DM in the ileum; 2.88 g water/g DM in the caecum) at the 240 g potato hash silage inclusion level. The WHC of the digesta from the proximal colon decreased linearly (*p* < 0.05), while there was a linear increase (*p* < 0.05) in WHC of the digesta from the distal colon with increasing levels of potato hash silage.

## 4. Discussion

The utilisation of nutrients by pigs is influenced by the dynamic interaction between digestion and digesta TT in the gastrointestinal tract [20]. Understanding this interaction becomes particularly important when non-conventional, fibrous feedstuffs, such as potato hash silage, are incorporated into pig diets. The increasing interest in such ingredients has highlighted the need to clarify how the physicochemical properties of these feeds influence rate of passage and nutrient digestibility [7]. This need is even greater in indigenous, slow-growing pigs like the Windsnyer, in which the mechanisms underlying their reported ability to utilise fibrous feeds more efficiently than improved breeds remain unclear [7]. If differences in digesta TT explain this enhanced utilisation, such insights may contribute to the characterisation and conservation of these genotypes. Our study examined how increasing inclusion levels of potato hash silage affected ATTD, MRT, and digesta physicochemical characteristics. A significant correlation between MRT and digestibility of DM, OM, and CP suggests that TT plays a functional role in nutrient availability in Windsnyer pigs. Similar relationships have been noted by Kim et al. [21] and support the use of MRT as an indicator in digestibility studies.

Potato hash silage had high WHC, and this was reflected in the diets, which showed a linear increase in WHC with increasing silage inclusion. However, digesta WHC did not follow the same pattern; instead, it decreased linearly in the stomach and proximal colon and responded quadratically in the ileum and caecum. Since WHC is associated with the amount and physicochemical structure of dietary fibre [22], an increase in digesta WHC would normally be expected. The observed discrepancy suggests that the ensiling process, inclusion of absorbents, and particle size reduction may have altered the hydration behaviour of the fibre within the digestive tract. Ensiling partially solubilizes some NSP fractions while compacting others [23], which can shift the balance between soluble and insoluble fibres and reduce their ability to retain water once exposed to gastric acidity and mechanical shear. Furthermore, responses of WHC of the digesta differed across gut segments, supporting the observation that WHC behaviour shifts as digesta progresses through the gastrointestinal tract. The increase in distal colon WHC at 320–400 g/kg inclusion may reflect increased fermentation-associated breakdown of non-starch polysaccharides (NSP) into shorter chains, which can attract water and swell more readily. Although NSP was not quantified, the pattern is consistent with known fermentation effects on partially degraded fibre [24].

Dietary SWC also increased with higher potato hash silage inclusion, reflecting the contribution of resistant starch and hydrophilic NSP. In the digesta, however, SWC responses were location dependent. The quadratic response in the stomach may reflect the combined effects of gastric acidity and mechanical agitation on the swelling behaviour of ensiled, mixed-source fibre. As the matrix hydrates and begins to solubilize, competition for water among fibre particles, starch residues, and acids can produce non-linear swelling patterns [25]. The linear decrease in ileal SWC coincided with increasing ileal pH. SWC is strongly influenced by the hydrogen-bonding environment [26]; as pH rises, the electrostatic repulsion among fibre molecules decreases, reducing their ability to imbibe water. This pH–hydration relationship has been well documented for NSP [13]. In the colon, SWC did not differ across treatments despite decreasing pH. This discrepancy between ileum and colon likely reflects the much higher load of fermentation end-products in the colon, where microbial biomass, mucus accumulation, and short-chain fatty acids interact with fibre matrices in ways that dampen SWC differences that were evident upstream [27].

The quadratic decrease in MRT with increasing silage inclusion indicates a complex relationship between fibre level, diet bulkiness, and digesta flow. Although the direction differed from our initial hypothesis, it aligns with established effects of high dietary NDF and ADF on reducing digesta viscosity and increasing luminal bulk, thereby accelerating passage rate [22]. When potato hash was combined with maize cobs for ensiling, the resulting material was highly bulky and low in soluble carbohydrates, conditions that promote faster flow by reducing digesta cohesiveness and increasing mechanical stimulation of gut motility [28]. Thus, the reduction in MRT is biologically plausible and consistent with the reported increases in faecal output at higher inclusion levels. A shortened MRT can limit microbial exposure time [29], which may partly explain the decline in nutrient digestibility at the highest silage inclusions. This interaction between TT and fermentation potential is important for understanding how Windsnyer pigs balance fibre utilisation with passage constraints.

The quadratic improvement in ATTD of DM, OM, CP, and NDF up to moderate inclusion levels demonstrates that Windsnyer pigs can effectively utilise potato hash silage before digestibility declines at higher inclusion levels. This pattern mirrors previous findings for maize cob silage in the same genotype [7]. Ensiling likely enhanced fibre accessibility through partial depolymerization and acid hydrolysis, supporting the higher digestibility at lower inclusion levels. At high levels, however, increased fibre bulk, reduced viscosity, elevated glycoalkaloid content, and shortened MRT may combine to limit nutrient exposure to digestive enzymes, leading to reduced digestibility. The linear increase in stomach weight with higher silage inclusion likely reflects increased dietary fibre and associated muscular adaptations from greater peristaltic workload [30]. Colon weights also increased, consistent with fermentation-driven epithelial proliferation and greater digesta mass in the hindgut. These findings align with previous work demonstrating that dietary fibre can induce morphological responses in the gastrointestinal tract [13].

## 5. Conclusions

The positive correlation between rate of passage and nutrient digestibility reiterates that these dynamic processes are interdependent and should be considered when evaluating non-conventional feeds. The positive quadratic response in ATTD of nutrients and feed intake with increasing inclusion levels of potato hash silage indicates that Windsnyer pigs can effectively utilise fibrous feedstuffs up to the 240 g/kg inclusion level, beyond which nutrient digestibility and feed intake decline. Furthermore, increasing inclusion levels of potato hash silage resulted in a linear decrease in growth performance parameters, reflecting reduced nutrient availability at higher fibre contents. Variations in the physicochemical properties of the digesta also corresponded with these changes, highlighting their role in modulating nutrient utilisation and rate of passage.

## Figures and Tables

**Table 1:** Ingredients of experimental diets used for growing Windsnyer pigs.

Ingredient	Inclusion Levels of Potato Hash Silage (g/kg Dry Matter)					
	0	80	160	240	320	400
Yellow maize	693	612	558	487	410	328
Soybean meal (48% CP)	160	167	170	186	190	200
Potato hash silage	0	80	160	240	320	400
Wheat bran	86	75	50	25	20	10
Molasses	20	20	20	20	20	20
Limestone	20	21	17.7	16.8	15.9	15
Monocalcium phosphate	3.5	4.8	5.9	7.2	8.9	9.7
Sunflower oil	5	4.8	4.7	4.5	4	4
Salt	4	4	4	4	4	4
_L_-Lysine. hcl	4	4.6	4.5	3.7	3.7	3.8
_DL_-Methionine	0.5	1.6	1.1	1.0	1.0	1.0
_L_-Threonine	1.0	1.5	1.2	1.0	1.0	1.1
Vitamin-mineral premix ^(1)^	4	4	4	4	4	4

^(1)^ Provides (per kg of dry matter of diet): vitamin A, 4.8 mg; vitamin D_3_, 0.09 mg; vitamin E, 50 mg; vitamin K_3_ (43%), 1.0 mg; vitamin B_1_, 1.6 mg; vitamin B_2_, 2.6 mg; niacin (99.5%), 33.6 mg; vitamin B_12_, 0.01 mg; vitamin B_6_ 98%, 2.0 mg; choline (chloride 60%), 121 mg; folic acid (96% pure), 0.48 mg; biotin, 0.18 mg; calcium pantothenate (98%), 5.2 mg; zinc balitracin, 90.0 mg; manganese sulphate, 120.0 mg; zinc, 100 mg; copper, 8 mg; potassium iodide (Iodine 76.45%), 0.4 mg; cobalt sulphate, 0.2 mg; ferrous sulphate, 100.0 mg and selenium, 0.32 mg on dolomite carrier.

**Table 2:** Chemical and physicochemical properties of potato hash silage and experimental diets (*n* = 3) ^(1)^.

Item	Potato Hash Silage	Potato Hash Silage Inclusion Levels (g/kg DM)					
		0	80	160	240	320	400
Dry matter (g/kg)	337	886	845	801	744	730	662
Gross energy (MJ/kg DM ^(2)^)	17.4	18.4	18.7	18.9	18.9	18.8	18.4
Crude protein (g/kg DM)	132	162	156	154	151	148	141
Ether extract (g/kg DM)	12.0	74.2	72.0	64.0	56.0	49.7	43.0
Ash (g/kg DM)	39.4	47.8	55.7	60.3	65.9	70.9	74.2
Calcium (g/kg DM)	-	6.86	7.03	7.18	7.49	7.92	8.53
Phosphorous (g/kg DM)	-	3.64	3.77	3.82	3.92	4.43	5.12
Neutral detergent fibre (g/kg DM)	633	343	344	373	386	396	407
Acid detergent fibre (g/kg DM)	335	80.5	85.1	94.5	98.9	103	108
Acid detergent lignin (g/kg DM)	324	37.3	40.8	48.7	56.2	60.3	64.5
Physical properties							
Bulk density (g/mL)	1.14	1.50	1.50	1.47	1.35	1.33	1.24
Swelling capacity (mL/g)	4.85	3.13	3.20	3.33	3.55	3.56	3.70
Water holding capacity (g_water_/g_feed_ DM)	8.8	3.67	3.87	4.76	5.27	5.34	5.45

^(1)^ Number of replications. ^(2)^ DM, dry matter.

**Table 3:** Pearson’s correlation coefficients between nutrient digestibility and rate of digesta passage.

Component	MRT	DMD	OMD	CPD	ADFD	NDFD
TT	0.03 ^ns^	0.49 **	0.51 **	0.56 **	0.19 ^ns^	0.52 *
MRT	-	0.39 *	0.35 *	0.34 *	0.17 ^ns^	0.31 ^ns^
DMD	-	-	0.53 **	0.52 **	0.04 ^ns^	0.35 *
OMD	-	-	-	0.79 **	0.22 ^ns^	0.74 ***
CPD	-	-	-	-	0.01 ^ns^	0.73 ***
ADFD	-	-	-	-	-	0.31 ^ns^

ADFD, acid detergent fibre digestibility; CPD, crude protein digestibility; DMD, dry matter digestibility; MRT, mean retention time; NDFD, neutral detergent fibre digestibility; OMD, organic matter digestibility; TT, transit time. *** *p* < 0.001; ** *p* < 0.01; * *p* < 0.05; ns, not significant.

**Table 4:** Effects of dietary levels of potato hash silage on nutrient digestibility and rate of digesta passage in growing Windsnyer pigs.

Variables	Means of Potato Hash Silage Inclusion Level (g/kg DM)						SEM	Orthogonal Polynomial Contrast (*p*-Value) ^(1)^	
	0	80	160	240	320	400		Linear	Quadratic
DMD	0.87	0.88	0.91	0.92	0.87	0.85	0.02	0.195	0.001
OMD	0.91	0.92	0.92	0.93	0.90	0.89	0.03	0.825	0.002
CPD	0.80	0.82	0.84	0.84	0.80	0.79	0.04	0.890	0.000
NDFD	0.68	0.70	0.72	0.75	0.64	0.63	0.05	0.487	0.042
ADFD	0.45	0.43	0.42	0.43	0.42	0.43	0.04	0.897	0.693
Transit time (h)	17.6	17.4	17.2	17.1	16.6	15.6	0.45	0.003	0.246
Mean retention time (h)	23.7	23.8	21.5	20.0	18.3	18.1	2.91	0.047	0.007
Faecal scores ^(2)^	2.92	2.52	2.87	3.05	4.49	3.08	0.69	0.546	0.143
Fresh faecal weight (kg)	4.71	5.14	5.67	6.23	7.98	8.55	0.59	0.000	0.275
Dry faecal weight (kg)	1.35	1.58	1.89	2.23	2.67	3.12	0.37	0.004	0.367

DFD, acid detergent fibre digestibility; CPD, crude protein digestibility; DM, dry matter; DMD, dry matter digestibility; NDFD, neutral detergent fibre digestibility; OMD, organic matter digestibility; SEM, standard error of the mean. ^(1)^ Cubic response was not observed. ^(2)^ Faecal scores 1 = watery, 2 = semi-watery, 3 = normal, 4 = semi-dry, 5 = dry.

**Table 5:** Effects of dietary levels of potato hash silage on feed intake and growth performance in growing Windsnyer pigs.

Variables	Means of Potato Hash Silage Inclusion Level (g/kg DM)						SEM	Orthogonal Polynomial Contrast (*p*-Value) ^(1)^	
	0	80	160	240	320	400		Linear	Quadratic
Initial body weight (kg)	20.8	20.2	21.1	23.7	23.2	23.8	1.56	0.157	0.278
Daily feed intake (kg)	1.52	1.65	1.74	1.92	1.68	1.56	0.07	0.124	0.005
Average daily gain (kg)	0.48	0.46	0.45	0.42	0.35	0.31	0.04	0.000	0.164
Gain-to-feed ratio	0.31	0.28	0.25	0.23	0.21	0.19	0.03	0.000	0.214
Final body weight	35.2	36.8	37.4	38.9	36.9	36.1	4.33	0.154	0.005

DM, dry matter; SEM, standard error of the mean. ^(1)^ Cubic response was not observed.

**Table 6:** Effects of dietary levels of potato hash silage on scaled digesta and compartment weights (g/kg BW/d).

Variable	Means of Potato Hash Silage Inclusion Level (g/kg DM)						SEM	Polynomial Orthogonal Contrast (*p*-Value) ^(1)^	
	0	80	160	240	320	400		Linear	Quadratic
Compartments weights									
Scaled stomach	9.4	11.6	10.3	11.6	11.6	14.7	0.75	0.007	0.466
Scaled small intestines	17.0	21.5	20.7	16.8	19.9	22.2	1.85	0.182	0.212
Scaled caecum	1.6	3.01	1.81	2.91	2.02	3.22	0.47	0.909	0.114
Scaled colon	11.8	15.3	16.5	17.3	14.1	19.6	1.58	0.041	0.102
Digesta weights									
Scaled stomach	25.3	17.5	18.5	20.0	32.3	22.1	4.37	0.246	0.551
Scaled small intestines	8.74	7.18	9.12	8.76	12.2	9.02	1.89	0.413	0.138
Scaled caecum	14.9	7.59	11.9	10.3	16.9	9.4	2.39	0.547	0.245
Scaled colon	11.4	12.9	13.2	17.0	16.9	27.6	2.72	0.000	0.267

^(1)^ Cubic response was not observed. BW, body weight; DM, dry matter; SEM, standard error of the mean.

**Table 7:** Effects of dietary levels of potato hash silage on digesta pH in the ileum, caecum, proximal colon, and distal colon.

Variable	Means of Potato Hash Silage Inclusion Level (g/kg DM)						SEM	Polynomial Orthogonal Contrast (*p*-Value) ^(1)^	
	0	80	160	240	320	400		Linear	Quadratic
Stomach digesta	3.5	3.5	3.4	4.2	3.5	3.4	0.23	0.246	0.092
Ileum digesta	5.9	6.2	6.4	6.5	6.5	6.6	0.13	0.000	0.147
Caecum digesta	6.1	6.1	6.3	6.4	6.1	6.3	0.11	0.526	0.361
Proximal colon digesta	6.4	6.5	6.6	6.3	6.2	6.6	0.15	0.215	0.114
Distal colon digesta	6.8	6.7	6.6	6.4	6.4	6.3	0.11	0.004	0.264

DM, Dry matter; SEM, standard error of the mean. ^(1)^ Cubic response was not observed.

**Table 8:** Swelling capacity and water holding capacity of the digesta from gut compartments of Windsnyer pigs fed potato hash silage.

Variable	Means of Potato Hash Silage Inclusion Level (g/kg DM)						SEM	Orthogonal Polynomial Contrast (*p*-Value) ^(1)^	
	0	80	160	240	320	400		Linear	Quadratic
Swelling capacity (mL/g DM)									
Stomach digesta	3.16	3.24	3.45	3.45	3.33	3.59	0.04	0.365	0.031
Ileum digesta	3.28	2.78	2.68	2.58	2.60	2.45	0.7	0.000	0.092
Caecum digesta	2.33	2.52	2.67	2.75	2.28	2.76	0.08	0.024	0.116
Proximal colon digesta	3.13	2.85	2.95	2.85	3.12	2.98	0.11	0.145	0.694
Distal colon digesta	2.45	2.38	2.47	2.45	2.42	2.40	1.61	0.324	0.106
Water holding capacity (g water/g DM)									
Stomach digesta	2.68	2.59	3.47	2.46	2.46	2.39	0.36	0.000	0.098
Ileum digesta	4.02	3.77	3.62	3.10	3.33	3.62	0.12	0.294	0.000
Caecum digesta	3.52	3.42	3.33	2.88	3.22	3.55	0.10	0.111	0.005
Proximal colon digesta	2.87	2.78	2.81	2.53	2.42	2.40	0.10	0.000	0.542
Distal colon digesta	2.28	2.57	2.63	2.47	3.03	3.15	0.08	0.000	0.347

DM, dry matter; SEM, standard error of the mean. ^(1)^ There was no cubic response observed.

## Data Availability

The raw data supporting the conclusions of this article will be made available by the authors on request.

## Abbreviations

The following abbreviations are used in this manuscript:

DM	Dry Matter
CP	Crude Protein
NDF	Neutral Detergent Fibre
ADF	Acid Detergent Fibre
ATTD	Apparent Total Tract Digestibility
DMD	Dry Matter Digestibility
OMD	Organic Matter Digestibility
CPD	Crude Protein Digestibility
NDFD	Neutral Detergent Fibre Digestibility
ADFD	Acid Detergent Fibre Digestibility
MRT	Mean Retention Time
TT	Transit Time
WHC	Water Holding Capacity
SWC	Swelling Capacity

## References

[1] Halimani T.E., Muchadeyi F.C., Chimonyo M., Dzama K. (2013). Opportunities for conservation and utilisation of local pig breeds in low-input production systems in Zimbabwe and South Africa. Trop. Anim. Health Prod..

[2] Thabethe F., Hlatini V.A., de Almeida A.M., Chimonyo M. (2022). Growth performance of South African Windsnyer pigs to the dietary inclusion of Amarula oil cake. Trop. Anim. Health Prod..

[3] Munzhelele P., Oguttu J., Fasanmi O.G., Fasina F.O. (2017). Production constraints of smallholder pig farms in agroecological zones of Mpumalanga, South Africa. Trop. Anim. Health Prod..

[4] Bovula N., Ncobela C.N., Pilane C.M., Nedambale T.L., Chimonyo M. (2021). Growth performance and fertility of Windsnyer boars supplemented with α-tocopherol. Trop. Anim. Health Prod..

[5] Ncobela C.N., Kanengoni A.T., Hlatini V.A., Thomas R.S., Chimonyo M. (2017). A review of the utility of potato by-products as a feed resource for smallholder pig production. Anim. Feed Sci. Technol..

[6] Nkosi B.D., Meeske R. (2010). Effects of ensiling totally mixed potato hash ration with or without a heterofermentative bacterial inoculant on silage fermentation, aerobic stability, growth performance and digestibility in lambs. Anim. Feed Sci. Technol..

[7] Kanengoni A.T., Chimonyo M., Ndimba B.K., Dzama K. (2015). Feed preference, nutrient digestibility and colon 572 volatile fatty acid production in growing South African Windsnyer-type indigenous pigs and Large White × 573 Landrace crosses fed diets containing ensiled maize cobs. Livest. Sci..

[8] Ngoc T.T., Len N.T., Lindberg J.E. (2013). Impact of fibre intake and fibre source on digestibility, gut development, retention time and growth performance of indigenous and exotic pigs. Animal.

[9] Carter N.A., Dewey C.E., Thomas L.F., Lukuyu B., Grace B., de Lange C. (2016). Nutrient requirements and low-cost balanced diets, based on seasonally available local feedstuffs, for local pigs on smallholder farms in Western Kenya. Trop. Anim. Health Prod..

[10] (2018). Winfeed: Feed Formulation Software. The Cheapest Least Cost Feed Formulation Software. https://www.winfeed.com.

[11] Lallès J.P., Delval E., Poncet C. (1991). Mean retention time of dietary residues within the gastrointestinal tract of the young ruminant: A comparison of non-compartmental (algebraic) and compartmental (modelling) estimation methods. Anim. Feed Sci. Technol..

[12] Faichney G.J., McDonald I.W., Warber A.C.I. (1975). The use of markers to partition digestion within the gastrointestinal tract of ruminants. Digestion and Metabolism in the Ruminant.

[13] Wate A., Zindove T.J., Chimonyo M. (2014). Effects of feeding incremental levels of maize cob meal on physicochemical properties of bulkiness in digesta in growing pigs. Livest. Sci..

[14] AOAC (2005). Official Methods of Analysis.

[15] Van Soest P.V., Robertson J.B., Lewis B.A. (1991). Methods for dietary fiber, neutral detergent fiber, and nonstarch polysaccharides in relation to animal nutrition. J. Dairy Sci..

[16] Kyriazakis I., Emmans G.C. (1995). The voluntary feed intake of pigs given feeds based on wheat bran, dried citrus pulp and grass meal, in relation to measurements of feed bulk. Br. J. Nutr..

[17] Whittemore E.C., Emmans G.C., Kyriazakis I. (2003). The relationship between live weight and the intake of bulky foods in pigs. Anim. Sci..

[18] Canibe N., Bach Knudsen K. (2002). Degradation and physicochemical changes of barley and pea fibre along the gastrointestinal tract of pigs. J. Sci. Food Agric..

[19] SAS (2008). SAS User’s Guide: Statistics.

[20] Wilfart A., Montagne L., Simmins H., Noblet J., van Milgen J. (2007). Digesta transit in different segments of the gastrointestinal tract of pigs as affected by insoluble fibre supplied by wheat bran. Br. J. Nutr..

[21] Kim B.G., Lindemann M.D., Cromwell G.L., Balfagon A., Agudelo J.H. (2007). The correlation between passage rate of digesta and dry matter digestibility in various stages of swine. Livest. Sci..

[22] Schop M., Jansman A.J., de Vries S., Gerrits W.J. (2020). Increased diet viscosity by oat β-glucans decreases the passage rate of liquids in the stomach and affects digesta physicochemical properties in growing pigs. Animal.

[23] Kanengoni A.T., Chimonyo M., Ndimba B.K., Dzama K. (2015). Potential of using maize cobs in pig diets—A review. Asian-Australas. J. Anim. Sci..

[24] Bachmann M., Michel S., Greef J.M., Zeyner A. (2021). Fermentation characteristics and in vitro digestibility of fibers and fiber-rich byproducts used for the feeding of pigs. Animals.

[25] Zijlstra R.T., Jha R., Woodward A.D., Fouhse J., Van Kempen T.A. (2012). Starch and fiber properties affect their kinetics of digestion and thereby digestive physiology in pigs. J. Anim. Sci..

[26] Zhao Y., Dang X., Du H., Wang D., Zhang J., Liu R., Ge Z., Sun Z., Zhong Q. (2024). Understanding the impact of extrusion treatment on cereals: Insights from alterations in starch physicochemical properties and in vitro digestion kinetics. Animals.

[27] Capuano E. (2017). The behavior of dietary fiber in the gastrointestinal tract determines its physiological effect. Crit. Rev. Food Sci. Nutr..

[28] Meldrum O.W., Yakubov G.E. (2025). Journey of dietary fiber along the gastrointestinal tract: Role of physical interactions, mucus, and biochemical transformations. Crit. Rev. Food Sci. Nutr..

[29] Gardiner G.E., Metzler-Zebeli B.U., Lawlor P.G. (2020). Impact of intestinal microbiota on growth and feed efficiency in pigs: A review. Microorganisms.

[30] Chassé É., Guay F., Bach Knudsen K.E., Zijlstra R.T., Létourneau-Montminy M.P. (2021). Toward precise nutrient value of feed in growing pigs: Effect of meal size, frequency and dietary fibre on nutrient utilisation. Animals.

